# Relationships between Diet, Alcohol Preference, and Heart Disease and Type 2 Diabetes among Americans

**DOI:** 10.1371/journal.pone.0124351

**Published:** 2015-05-11

**Authors:** Michael K. Adjemian, Richard J. Volpe, Jennifer Adjemian

**Affiliations:** 1 Economic Research Service, United States Department of Agriculture, Washington, District of Columbia, United States of America; 2 Agribusiness Department, College of Agriculture, Food, and Environmental Sciences, California Polytechnic State University, San Luis Obispo, California, United States of America; 3 Epidemiology Unit, Laboratory of Clinical Infectious Diseases, National Institute of Allergy and Infectious Diseases, Bethesda, Maryland, United States of America; 4 United States Public Health Service Commissioned Corps, Rockville, Maryland, United States of America; University of Bologna, ITALY

## Abstract

Although excessive alcohol consumption is a recognized cause of morbidity and mortality, many studies have linked moderate alcohol consumption to improved cardiovascular health and a lower risk of Type 2 Diabetes (T2D). Self-reported alcohol and diet data used to generate these results suffer from measurement error due to recall bias. We estimate the effects of diet, alcohol, and lifestyle choices on the prevalence and incidence of cardiovascular disease and T2D among U.S. adults using a nationally representative cohort of households with scanner data representing their food-at-home, alcohol, and tobacco purchases from 2007-2010, and self-reported health surveys for the same study participants from 2010-2012. Multivariate regression models were used to identify significant associations among purchase data and lifestyle/demographic factors with disease prevalence in 2010, and with incidence of new disease from 2011-2012. After controlling for important confounders, respondents who purchased moderate levels of wine were 25% less likely than non-drinkers to report heart disease in 2010. However, no alcohol-related expenditure variables significantly affected the likelihood of reporting incident heart disease from 2011-2012. In contrast, many types of alcohol-related purchases were associated with a lower prevalence of T2D, and respondents who purchased the greatest volumes of wine or beer—but not liquor—were less likely to report being diagnosed with T2D in 2011-2012 than non-drinkers.

## Introduction

Excessive alcohol consumption is a well-recognized cause of morbidity [1] and mortality, accounting for 80,000 United States (U.S.) deaths annually [2]. However, many studies have linked moderate alcohol consumption to improved cardiovascular health [3] and a lower Type 2 Diabetes (T2D) risk [4], with few health benefits and even negative outcomes observed at lower and higher alcohol consumption levels. Yet, in these studies, it is often difficult to separate effects of alcohol use from other lifestyle-related factors on health outcomes, including an individual’s behavior and diet [5]. Given the known hazards associated with drinking greater amounts of alcohol, it is critical to control for these potentially confounding lifestyle factors to provide careful public health recommendations regarding alcohol consumption [6].

Some of the cardio-protective health effects attributed to alcohol may be a product of lifestyle-related differences that exist between those who choose to drink moderate levels of alcohol versus those who drink excessively or abstain. For instance, moderate drinkers have been shown to be less likely than non-drinkers and heavy drinkers to exhibit a wide variety of factors linked to heart disease, such as physical inactivity [5, 7]. In addition, the preferred alcohol-type consumed has also been shown to affect health outcomes. Wine in particular has been identified as perhaps the most beneficial alcohol choice, since rates of cardiovascular disease and/or related deaths appear to be lowest for wine drinkers [8, 9]. However, wine drinkers have also been shown to eat healthier, exercise more, and have higher intelligence quotients, income levels, and more education then even their drinking peers [10–15]. Nevertheless, randomized clinical studies have identified a favorable effect of alcohol on several important biomarkers for heart disease and T2D [16–20].

Many observational studies have attempted to control for some of these confounders and have found no significant reduction in estimated benefits of alcohol consumption. But virtually all have relied on self-reported recollections of study subjects through food diaries, which are subject to substantial measurement error [21]. In many cases, the interval between entries in a given food diary, as well as the recollection period required of subjects, can range up to a period of years [22, 23]. Household food and alcohol transactions via scanner data are far more comprehensive [11, 24], providing a powerful means to observe dietary and drinking choices [6]. Using a nationally representative cohort of households whose food-at-home, alcohol, and tobacco purchases are captured over a four-year period, and associating them with up to three years of self-reported health surveys for the same study participants, we report on the relationships between diet, alcohol, and lifestyle choices with the prevalence and incidence of cardiovascular disease and T2D among U.S. adults.

## Methods

### Survey Population

We obtained National Consumer Panel (NCP) data from 2007–2012. The NCP, a joint venture of the Nielsen Corporation and Information Resources, Inc. (IRI), is a large and nationally representative dataset of U.S. households measuring retail purchases. Participating households are provided scanner equipment and instructions for its use and reporting, and are compensated for their ongoing participation in the panel. Panelists are selected to produce a nationally representative sample based on household size, household head’s age, income, race/ethnicity, presence of children, and urban/rural location.

### Data Collection

NCP households are instructed to record all retail purchases by scanning universal product codes (UPCs) or supplemental scan codes for non-UPCed items. Household demographics and socioeconomic status are collected and maintained, and annual “Medprofiler” health surveys were conducted by IRI beginning in 2010 from a subset of NCP households. These health surveys collected data on each household member’s self-reported age, weight, height, health concerns, exercise, stress-level, and presence of selected health conditions, as reported by the household head. This study used purchase data obtained from 2007–2010 and health data reported from 2010–2012 for individuals aged ≥21 years.

### Dietary Healthfulness

Dietary health scores were calculated using both Healthshare and USDAscore. Healthshare reports the proportion of purchases categorized as foods recommended for increased consumption by the U.S. Department of Agriculture’s (USDA) and U.S. Department of Health and Human Services’ 2010 Dietary Guidelines for Americans [25–27]. USDAscore—equivalent to Volpe and Okrent’s USDAScore1—is calculated using the same purchases but modified based on the age and number of household members, and their adherence to food expenditure recommendations by the USDA Center for Nutritional Policy and Promotion [25, 27].

### Alcohol and tobacco consumption

Alcohol-consumption was calculated based on a household’s per-capita purchased drinks for at-home consumption [28]. Volume was converted into number of drinks according to the following single drink definition: 12.5oz (370mL) of beer, 5oz (148mL) of wine, and 1.5oz (44mL) of spirits. Drinking households were then stratified into thirds based on average annual per-capita alcohol purchases. Households without any purchased alcohol were labeled non-drinkers. Alcohol-type preference was defined by the type of alcohol representing the majority of household expenditures. Households were classified by their alcohol preference in conjunction with expenditure level (e.g., "beer 3" represents households with alcohol expenditures in the top-third of sampled homes, with the majority of its expenditures on beer). We evaluated the sensitivity of the type-preference definition by varying the amount of other types of alcohol a preferring household could purchase, up to defining type X drinkers as those who purchasing only type X alcohol. Households with any tobacco purchases were considered “tobacco users”. Drinker types were compared to non-drinkers using two-sided t-tests for group means.

### Health Outcomes

Using self-reported health data from 2010–2012, we sought to identify factors associated with incident and prevalent heart disease and/or T2D. Prevalence was assessed using 2010 survey data, where heart disease included those reporting “heart problems not including a heart attack” and/or “heart attack” and T2D included those reporting that diagnosis. To assess incidence, survey participants not reporting the condition in 2010 were included and followed to determine whether they later reported it in 2011–2012. Other health conditions included as possible predictors included high blood pressure and cholesterol. Self-reported stress and activity levels were also assessed; all respondents reporting being “very” or “somewhat” concerned over stress-levels were labeled positive for stress concerns, and those reporting exercising or being active ≥20 minutes per day on “most” or “some” days were labeled as such. Body mass index (BMI) was calculated using self-reported height and weight.

### Statistical Analysis

Dietary, demographic, socioeconomic and health-related factors were included for analysis to assess risks for incident and prevalent heart disease and T2D. For each condition, logistic regression models were used to estimate factors predictive of prevalent and incident heart disease and T2D. Univariate analyses were conducted to identify significant (p<0.05) unadjusted variables, which were then applied in a multivariate model to obtain adjusted odds ratios (aOR); 95% confidence intervals (CI) were constructed around all estimates. All multivariate models were assessed to ensure adequate ratios of events per parameter [29]. For brevity, ORs for heart disease/T2D prevalence among 2010 survey participants are reported as “OR_2010_”, while ORs for incidence from 2011–2012 are represented by “OR_2012_”. To account for potential confounders, factors identified as predictive for at least one condition via univariate analysis or due to biological relevance were retained in all multivariate models. All alcohol-related variables were also retained to demonstrate their effects. Analyses were conducted using STATA [30].

## Results

### Study Population Demographics and Health Profile

In 2010, 59,545 (82%) of 72,878 enlisted NCP households provided sufficient food purchase and demographic information for analysis. Of these, 49,377 participants in 26,719 (45%) households were ≥21 years old and returned the 2010 health survey; descriptive statistics for respondents are displayed in Table 1. Among included respondents, 46% were male and 86% were White and/or Hispanic, 7% Black, 3% Asian, and 4% reported belonging to another race/ethnicity. The median age of included respondents was 55 (range: 21–102) and median annual household income and education years of the household head were $55,000 (range: $6,500-$225,000) and 16 (range: 8–18), respectively. Most participants lived in the Midwest (37%) and South (27%), while the remainder resided in the West (19%) and Northeast (17%); 21% of included respondents lived in rural areas.

**Table 1:** Variable Means for the 2010 MedProfiler Survey Population Households, Stratified by Alcohol Type Preference^a^.

	All Participants	Wine	Beer	Liquor	Non-Drinkers
	*N = 49*,*377*	*N = 15*,*452*	*N = 11*,*317*	*N = 9*,*731*	*N = 12*,*894*
**High Cholesterol**	36.1	37.6***	34.3*	36.6*	35.6
**High Blood Pressure**	33.4	33.8	31.1***	34.8	34
**Heart Disease** ^**b**^	9.8	9.8*	9.1***	9.9	10.4
**Type 2 Diabetes**	10.8	9.7***	10.1***	10.4***	13
**Female**	54	55	52***	53***	55
**Age (years)**	54	56***	52***	54***	53
**White or Hispanic**	86	86***	86***	85	84
**Black**	7	6***	7***	9	8
**Asian**	3	4**	3***	2***	3
**Other Race**	4	4	4***	4	4
**Married**	72	73***	76***	70	69
**Single**	11	11***	10***	12	12
**Children present?**	23	19***	27*	20***	26
**Midwest**	37	39	35***	30***	40
**South**	27	24	30***	32***	24
**West**	19	22***	17**	22***	16
**Northeast**	17	15***	17***	17***	20
**Rural**	21	18***	23	21***	23
**Body-Mass Index (Points)**	28.8	27.9***	28.8***	29.1***	29.5
**Concerned About Stress**	72	70***	73	71***	73
**Exercise Most Days**	37	40***	36*	36	35
**Exercise Some Days**	36	36	35	36	36
**Household Income ($K)**	64	73***	62*	66***	55
**Healthshare**	32.3	35.1***	30.5***	32.1***	30.9
**USDAscore (Points)**	7.8	8.3***	7.8***	7.9***	7.3
**Drinker 1** ^**c**^	24	34	39	24	0
**Drinker 2** ^**c**^	24	31	35	35	0
**Drinker 3** ^**c**^	25	35	27	41	0
**Smoker**	25	21	33***	31***	20

Notes: Unless otherwise indicated, all values in the table represent percentages. The "All Participants" total is slightly smaller than the sum of the population sizes for each alcohol type category, since a handful of included respondents were classified in multiple categories because spending on multiple alcohol types was equivalent. Healthshare and USDAscore are aggregate average dietary index scores, based on a household’s scanned purchases from 2007–2010.

^a^Alcohol type preference was defined as the type of alcohol on which the participant's household spent the most from 2007–2010. “Non-drinkers” recorded no alcohol purchases in the sample.

^b^Heart disease includes respondents who reported "heart problems" as well as heart attacks. The significance of the results in the table is unchanged when only "heart problems” are considered.

^c^Drinker levels are classified in thirds, increasing with the household's average per-capita alcohol expenditures.

*** *P*<0.01

** *P*<0.05

* *P*<0.1

The mean BMI score of included respondents was 29 (range: 15–76), while the average dietary health score of households surveyed was 32 (range: 2–99) as reported by Healthshare, and 8 (range: 1–33) as represented by USDAscore. Stress was reported as a concern by 72% of respondents; 37% claimed to exercise on most days. Among included respondents, 36% reported high cholesterol, 33% high blood pressure, 10% heart disease, and 11% T2D in 2010. Of those 2010 survey respondents who did not report heart disease or T2D, 25,196 (57%) and 24,784 (56%) submitted follow-up surveys from 2011–2012, respectively. From these, 2,373 (9%) newly reported heart disease and 1,487 (6%) T2D from 2011–2012.

### Comparisons of Drinkers and Non-Drinkers

According to Table 1, one-quarter of participants lived in households without any alcohol purchases. When classified by the majority of household alcohol purchases, 31% were labeled wine drinkers, 23% beer drinkers, and 20% liquor drinkers; 25% of respondents lived in households purchasing tobacco products. Greater percentages of participants in wine drinking homes (40%) than non-drinking homes (35%) reported exercising on most days. Wine ($73k) and liquor ($66k) drinkers had higher mean income levels than non-drinkers ($55k). Males household heads constituted greater percentages of beer (48%) and liquor drinkers (47%) than non-drinkers (45%). Wine (mean age = 56 years) and liquor drinkers (54 years) were significantly older, and beer drinkers (52 years) younger, than non-drinkers (53). The mean BMI for each drinker group (27.9, 28.8, and 29.1 for wine, beer, and liquor, respectively) was significantly lower than non-drinkers (29.5). Wine (70%) and liquor (71%) drinkers were less frequently concerned about stress than non-drinkers (73%). According to both measures used to account for dietary health, wine and liquor drinkers had healthier diets than non-drinkers; beer drinkers had a slightly lower mean Healthshare score but higher mean USDAscore than non-drinkers.

### Predictors for Self-reported Heart Disease and T2D

#### Associations with alcohol

Factors associated with prevalent and incident heart disease and T2D from logistic regression analyses are shown in Tables 2 and 3, respectively. In univariate models, several alcohol consumption variables were associated with lower prevalence and incidence of heart disease (e.g., for Beer 3, OR_2010_ = 0.8, CI: 0.7–0.9); on the other hand, heavy liquor consumption was associated with an increased risk for prevalent (OR_2010_ = 1.1, CI: 1.1–1.3) and incident (OR_2012_ = 1.2, CI: 1.1–1.4) heart disease. However, in adjusted models, only respondents that purchased moderate alcohol volume-levels, and that demonstrated a preference for wine (i.e., “wine 2” homes), were less likely than others to report heart disease in 2010 (aOR_2010_ = 0.8, CI: 0.7–0.9). No alcohol expenditure variables significantly affected the likelihood of reporting incident heart disease from 2011–2012 in the adjusted model.

**Table 2:** Association between Sociodemographic, Health, and Lifestyle Factors with the Prevalence and Incidence of Heart Disease^+^, 2010–2012.

	Risk of having Heart Disease in 2010 (N = 4,841)				Risk of reporting Heart Disease in 2011–2012,			
					after not reporting it in the 2010 survey (N = 2,373)			
	*OR*_*2010*_	*95% C*.*I*.	*aOR*_*2010*_	*95% C*.*I*.	*OR*_*2012*_	*95% C*.*I*.	*aOR*_*2012*_	*95% C*.*I*.
**High Cholesterol**	5.3***	(5.0–5.7)	2.4***	(2.2–2.6)	2.1***	(1.9–2.3)	1.3***	(1.2–1.5)
**High Blood Pressure**	6.0***	(5.6–6.5)	2.8***	(2.6–3.0)	2.4***	(2.2–2.6)	1.6***	(1.4–1.8)
**Type 2 Diabetes**	3.6***	(3.4–3.9)	1.6***	(1.4–1.7)	2.0***	(1.8–2.3)	1.2***	(1.1–1.4)
**Female**	0.6***	(0.5–0.6)	0.6***	(0.5–0.6)	0.8***	(0.8–0.9)	0.9***	(0.8–0.9)
**Age (in 10 yrs)**	1.9***	(1.8–1.9)	1.6***	(1.5–1.7)	1.5***	(1.5–1.6)	1.4***	(1.4–1.5)
**Body-Mass Index**	1.03***	(1.02–1.03)	1.0	(0.99–1.01)	1.02***	(1.01–1.03)	1.01***	(1.01–1.02)
**Concerned About Stress**	1	(0.9–1.1)	1.3***	(1.2–1.4)	1.0	(0.9–1.1)	1.2***	(1.1–1.3)
**Exercise Most Days**	0.77***	(0.7–0.8)	0.82***	(0.8–0.9)	0.84***	(0.8–0.92)	0.86**	(0.8–0.97)
**Exercise Some Days**	0.89***	(0.83–0.95)	0.86***	(0.8–0.9)	0.9	(0.9–1.0)	0.9**	(0.8–0.99)
**Income (in $10K)**	0.92***	(0.91–0.93)	0.96***	(0.95–0.97)	0.97***	(0.96–0.98)	1.0	(1.0–1.0)
**Historical Dietary Score** ^	1.0***	(1.0–1.0)	1.0	(1.0–1.0)	1.0**	(1.0–1.0)	1.0	(1.0–1.0)
**Wine 1**	1.1	(1.0–1.2)	1.0	(0.8–1.1)	1.0	(0.8–1.1)	0.9	(0.7–1.1)
**Wine 2**	0.9**	(0.8–0.9)	0.8***	(0.7–0.9)	1.1	(0.9–1.2)	1.0	(0.8–1.2)
**Wine 3**	1	(0.9–1.1)	0.9	(0.8–1.0)	1.0	(0.8–1.1)	0.9	(0.7–1.0)
**Beer 1**	1	(0.9–1.1)	0.9	(0.8–1.1)	1.0	(0.9–1.2)	0.9	(0.8–1.1)
**Beer 2**	0.9	(0.8–1.0)	1.0	(0.9–1.1)	0.8**	(0.7–0.9)	0.9	(0.7–1.1)
**Beer 3**	0.8***	(0.7–0.9)	0.9	(0.8–1.0)	0.9	(0.8–1.1)	0.9	(0.7–1.1)
**Liquor 1**	1	(0.8–1.1)	0.9	(0.8–1.1)	0.8	(0.7–1.1)	0.8	(0.6–1.0)
**Liquor 2**	0.9	(0.8–1.0)	1.0	(0.8–1.1)	1.0	(0.8–1.2)	1.0	(0.8–1.3)
**Liquor 3**	1.1**	(1.1–1.3)	0.9	(0.8–1.0)	1.2**	(1.1–1.4)	1.0	(0.9–1.2)
**Smoker**	1.2***	(1.1–1.3)	1.3***	(1.2–1.4)	1.2***	(1.1–1.4)	1.3***	(1.2–1.5)
**Respondents**	49,377				25,196			
**Pseudo R-squared**	-		19%		-		6%	

^+^Heart disease includes respondents who reported "heart problems" as well as heart attacks. The significance of the results in the table is unchanged when only "heart problems" is considered.

^^^The results in this table use Healthshare to represent diet score (virtually identical results are estimated with USDAscore).

OR = unadjusted Odds Ratio; aOR = adjusted Odds Ratio; C.I. = 95% confidence interval. Standard errors used to estimate confidence intervals are robust to clustering at the household level. Race, region, and marital status were controlled for in the multivariate analyses but are not presented in the table to conserve space. Levels of significance are represented as

*** *P*<0.01,

** *P*<0.05,

* *P*<0.1.

**Table 3:** Association between Sociodemographic, Health, and Lifestyle Factors with the Prevalence and Incidence of Type 2 Diabetes, 2010–2012.

	Risk of having T2D in 2010 (N = 5,323)				Risk of reporting T2D in 2011–2012,			
					after not reporting it in the 2010 survey (N = 1,487)			
	*OR*_*2010*_	*95% C*.*I*.	*aOR*_*2010*_	*95% C*.*I*.	*OR*_*2012*_	*95% C*.*I*.	*aOR*_*2012*_	*95% C*.*I*.
**Heart Disease**	3.6***	(3.4–3.9)	1.5***	(1.3–1.6)	2.1***	(1.8–2.4)	1.2**	(1.0–1.4)
**High Cholesterol**	5.5***	(5.1–5.8)	2.9***	(2.7–3.2)	2.0***	(1.8–2.3)	1.4***	(1.2–1.5)
**High Blood Pressure**	5.2***	(4.9–5.5)	2.0***	(1.9–2.2)	2.3***	(2.1–2.6)	1.4***	(1.2–1.6)
**Female**	0.7***	(0.7–0.8)	0.7***	(0.7–0.8)	0.9***	(0.8–0.9)	0.9**	(0.8–0.98)
**Age (in 10 yrs)**	1.5***	(1.5–1.6)	1.3***	(1.3–1.4)	1.3***	(1.3–1.4)	1.2***	(1.2–1.3)
**Body-Mass Index**	1.09***	(1.09–1.1)	1.08***	(1.08–1.09)	1.06***	(1.06–1.07)	1.05***	(1.05–1.06)
**Concerned About Stress**	1.0	(0.9–1.0)	1.1	(1.0–1.1)	1.1	(1.0–1.2)	1.2***	(1.1–1.3)
**Exercise Most Days**	0.6***	(0.6–0.6)	0.8***	(0.7–0.8)	0.7***	(0.6–0.8)	0.7***	(0.6–0.9)
**Exercise Some Days**	0.9***	(0.9–1.0)	0.9***	(0.8–0.9)	0.9*	(0.8–1.0)	0.8***	(0.7–0.9)
**Income (in $10K)**	0.93***	(0.93–0.94)	0.98***	(0.97–0.99)	0.94***	(0.92–0.96)	0.97***	(0.95–0.99)
**Historical Dietary Score^**	1.0***	(1.0–1.0)	1.01***	(1.0–1.01)	1.0	(1.0–1.0)	1.0	(1.0–1.0)
**Wine 1**	1.1**	(1.0–1.2)	0.9*	(0.8–1.0)	1.0	(0.9–1.2)	0.9	(0.7–1.1)
**Wine 2**	0.9	(0.9–1.1)	0.8***	(0.7–0.9)	0.9	(0.7–1.1)	0.8*	(0.6–1.0)
**Wine 3**	0.6***	(0.5–0.7)	0.5***	(0.5–0.6)	0.5***	(0.4–0.7)	0.5***	(0.4–0.7)
**Beer 1**	1.2***	(1.1–1.3)	0.9	(0.8–1.1)	1.2	(1.0–1.4)	1.0	(0.8–1.2)
**Beer 2**	0.9	(0.8–1.0)	0.9*	(0.8–1.0)	1.0	(0.8–1.3)	0.9	(0.7–1.2)
**Beer 3**	0.7***	(0.6–0.8)	0.6***	(0.5–0.7)	0.7**	(0.6–0.9)	0.7***	(0.5–0.9)
**Liquor 1**	1.3***	(1.1–1.4)	1.0	(0.9–1.2)	1.2	(0.9–1.5)	1.0	(0.7–1.3)
**Liquor 2**	0.9	(0.8–1.1)	0.8**	(0.7–0.9)	1.1	(0.9–1.3)	0.9	(0.7–1.2)
**Liquor 3**	0.8***	(0.7–0.9)	0.6***	(0.5–0.7)	1.1	(0.9–1.3)	0.9	(0.7–1.1)
**Smoker**	1.1*	(1.0–1.1)	1.1***	(1.1–1.2)	1.3***	(1.2–1.3)	1.3***	(1.2–1.5)
**Respondents**	49,377				24,784			
**Pseudo R-squared**	-		20%		-		7%	

^^^The results in this table use Healthshare to represent diet score. Virtually identical results are estimated when USDAscore is used.

OR = unadjusted Odds Ratio; aOR = adjusted Odds Ratio; C.I. = 95% confidence interval. Standard errors used to estimate confidence intervals are robust to clustering at the household level. Race, region, and marital status were controlled for in the multivariate analyses but are not presented in the table to conserve space. Levels of significance are represented as

*** *P*<0.01,

** *P*<0.05,

* *P*<0.1.

In univariate analyses, respondents with T2D were less likely to report drinking at high volume-levels and more likely to report drinking at low volume-levels, whatever their alcohol-type preference. Similarly, households that purchased the greatest alcohol volumes and demonstrated a preference for wine or beer, were 100% (OR_2012_ = 0.5, CI: 0.4–0.7) and 40% (OR_2012_ = 0.7, CI: 0.6–0.9) less likely, respectively, to report being diagnosed with T2D in 2011–2012 than non-drinkers. In multivariate analyses, all levels of wine drinkers and moderate to heavy beer or liquor drinkers were less likely to report T2D in 2010, and only high volume-level wine and beer households were less likely to report incident T2D in 2011–2012. Further analyses demonstrated robustness of these results to varying definitions of alcohol type preference, even when drinker types were defined only as those who bought a single type of alcohol.

#### Associations with other factors included in adjusted models

Respondents reporting high cholesterol in 2010 were 2.4 times more likely (CI: 2.2–2.6) to also report heart disease in 2010, and 30% more likely (CI: 1.2–1.5) to newly report heart disease from 2011–2012. For T2D, these values were 2.9 times (CI: 2.7–3.2) and 40% (CI: 1.2–1.5), respectively. Respondents reporting high blood pressure in 2010 were 2.8 times more likely (CI: 2.6–3.0) to also report heart disease in 2010, and 60% more likely (CI: 1.4–1.8) to newly report heart disease from 2011–2012. For T2D, these values were two times (CI: 1.9–2.2) and 40% (CI: 1.2–1.6) more likely, respectively. Reporting T2D in 2010 was associated with a 60% (CI: 1.4–1.7) higher likelihood of also reporting heart disease, and 20% (CI: 1.1–1.4) greater likelihood of first reporting heart disease from 2011–2012.

Women were less likely than men to have both heart disease (aOR_2010_ = 0.6, CI: 0.5–0.6) and T2D (aOR_2010_ = 0.7, CI: 0.7–0.8). They were also less likely to report either condition from 2011–2012 (aOR_2012_ = 0.9, CI: 0.8–0.9, and aOR_2012_ = 0.9, CI: 0.8–0.98, respectively). Each additional ten years of age was associated with an increased risk of 60% for reporting heart disease in 2010 (CI: 1.5–1.7) and 40% for 2011–2012 (CI:1.4–1.5), and also with an increased risk of 30% for reporting T2D (CI = 1.3–1.4) in 2010 and 20% in 2011–2012 (CI:1.2–1.3). Each additional one-unit increase in BMI was associated with an 8% chance of reporting T2D (CI: 1.08–1.09) in 2010. Each one-unit BMI increase also increased the risk of being diagnosed for the first time with heart disease by 1% (CI: 1.01–1.02) and with T2D by 5% (CI:1.05–1.06) in 2011–2012. Household diet scores were not significantly associated with reporting heart disease in 2010 according to the adjusted model, although people with T2D in 2010 had slightly healthier diet scores than those without T2D (aOR_2010_ = 1.01, CI: 1–1.01). Concerns over stress increased the likelihood of reporting prevalent heart disease (aOR_2010_ = 1.3, CI: 1.2–1.4), and incident heart disease (aOR2012 = 1.2; CI: 1.1–1.3) and T2D (aOR2012 = 1.2; CI: 1.1–1.3) from 2011–2012. Regular exercise reduced the risk of reporting both prevalent and incident heart disease (for exercise on some days, these values were aOR_2010_ = 0.86, CI: 0.8–0.9; aOR_2012_ = 0.9, CI: 0.8–0.99, respectively,) and T2D (aOR_2010_ = 0.9, CI: 0.8–0.9; aOR_2012_ = 0.8, CI: 0.7–0.9, respectively). The protective effect of exercise was even stronger, at the mean, for exercise on most days. Every additional $10K of household income decreased the risk of reporting both conditions by 2–3% (aOR_2010_ = 0.96, CI: 0.95–0.97 for heart disease; aOR_2010_ = 0.98, CI: 0.97–0.99 for T2D) in 2010, and of newly reporting T2D from 2011–2012 (aOR_2012_ = 0.97, CI: 0.95–0.99). Respondents from tobacco-using households were 30% more likely to report heart disease (CI: 1.2–1.4) and 10% more likely to report T2D (CI: 1.1–1.2) in 2010, and also 30% more likely to newly report either disease from 2011–2012 (CI: 1.2–1.5 for both).

## Discussion

This nationally representative population-based study is the first to use actual household-level food, alcohol, and tobacco purchases in combination with health outcomes data to evaluate their associations with having or developing heart disease and T2D. Although prior research demonstrated a cardio-protective effect of certain types of alcohol consumption [3], none classified drinkers based on actual purchases, and few adequately accounted for dietary factors that are thought to modify this relationship. Of those that do include diet, consumption data are self-reported and are collected infrequently [22, 31–36], offering a much narrower view, particularly given the associated problem of measurement error [37, 38]. These problems have prompted researchers to recognize the usefulness of actual purchase data to adequately address confounding [6, 11]. The findings of this study address a critical gap in the existing literature and serve to better elucidate the true relationship between alcohol consumption and heart disease and T2D, two increasingly prevalent and costly conditions in the United States.

Using NCP data over a four-year period, we did not find a significant link between any type and/or level of alcohol consumption and the odds of developing incident heart disease over the following two years, once critical confounders were controlled. Even though several alcohol consumption variables were protective for prevalent and incident heart disease in a univariate model, their significance disappeared once important confounders were included. The only type of alcohol consumption that remained significantly associated with lower heart disease prevalence in the multivariate model was a purchase preference for wine among respondents belonging to households with mid-level alcohol expenditures. On the other hand, household consumption of greater volumes of alcohol, regardless of type, was associated with lower odds of reporting prevalent T2D in 2010, while the highest levels of wine and beer purchases were protective for incident T2D over the next two years. These results demonstrate a beneficial role of alcohol with respect to diabetes, even after diet and lifestyle-related factors are taken into account.

Our results support that the relationship between alcohol consumption in general—and particularly with certain types of alcohol consumption—and heart disease is confounded by lifestyle and other factors. As others have shown [5, 7, 12–15], respondents in our study who live in households that purchase alcohol are more likely than non-drinkers to exhibit factors associated with a lower risk of heart disease. These respondents had lower BMIs, were less concerned about stress (for wine and liquor drinkers), exercised on most days (wine drinkers), had higher income (wine and liquor drinkers), and consumed healthier diets. Although our diet variables had small, though significant, estimated effects on certain health outcomes, they likely have a strong indirect effect via their operation through BMI. Smoking, on the other hand, unambiguously increased the risk for both heart disease and T2D, and some beer and liquor drinkers were more likely to reside in smoking households. At the same time, our multivariate models estimated that moderate wine drinking remained protective against heart disease, and several of our alcohol consumption variables remained associated with decreased risk of developing T2D. These findings further support the recent line of research identifying alcohol as a potential source of reduced T2D risk: alcohol may improve insulin sensitivity, increase high-density lipoprotein cholesterol and adiponectin, and confer anti-inflammatory effects [39].

Compared to the general U.S. population [40], respondents in our analysis were older (27% versus 16% >62 years old) and more frequently female (54% versus 51%). They were also more likely to be white (86% versus 75%) [41], and had higher median household income levels ($55,000 versus $50,000 per year) [42]. Our respondents were slightly less likely than the general U.S. population to report heart disease (10% versus 12%) and were more likely to report T2D (11% versus 9%), high blood pressure (33% versus 25%)[43], and high cholesterol (36% versus 14%), although this may be attributed to the older population present in our study [44].

Our study is limited by the fact that all purchase data are at the household-level, and only for at-home consumption. Thus, generalizing our results is constrained to the extent that diets differ among individuals within households and that away-from-home dietary behavior varies from at-home data. Further, our follow-up window is a two-year period, limiting our ability to identify all respondents who later develop heart disease and T2D. We also are unable to identify reasons for survey exit including mortality, which of course could include the sickest heart disease patients and therefore may make our population appear healthier than it actually is. Additionally, though we observe actual scanned purchases, we do not observe products that households failed to scan. Furthermore, all health outcomes in our study were self-reported; thus, one needs to have been sick enough to seek care and/or be aware of the diagnosis to report the condition. This would limit our ability to identify important differences between those with and without heart disease and T2D as some respondents who have the diseases may not be aware of them, and were therefore included here as a “non-diseased” group. Finally, some of our non-drinkers may represent “sick-quitters”, people who gave up drinking due to health problems, although this would bias our results in favor of protective effects associated with alcohol. Though NCP data are observational, their size and scope provides statistical robustness to tease apart the complicated effects of diet, alcohol preferences, and lifestyle factors on health.

## Conclusion

Our study is the first to combine observed household purchases with health outcomes to estimate relationships among dietary health, alcohol, and critical lifestyle factors. Moderate wine drinkers had lower rates of heart disease, though we were unable to establish a significant protective association of other types of alcohol consumption with heart disease. No alcohol type lowered the odds of first reporting heart disease over the following two-year period. In contrast, we found that several forms of alcohol consumption are associated with lower risks of both having and later reporting T2D.
